# Robotic versus laparoscopic left colectomy: a systematic review and meta-analysis

**DOI:** 10.1007/s00384-022-04194-8

**Published:** 2022-06-01

**Authors:** Leonardo Solaini, Antonio Bocchino, Andrea Avanzolini, Domenico Annunziata, Davide Cavaliere, Giorgio Ercolani

**Affiliations:** 1 Department of Medical and Surgical Sciences (DIMEC), University of Bologna, Bologna, Italy; 2 General and Oncologic Surgery, Morgagni-Pierantoni Hospital, Ausl Romagna, Forlì, Italy

**Keywords:** Left colectomy, Left hemicolectomy, Robotic surgery, Laparoscopy, Colorectal surgery

## Abstract

**Background:**

This study aimed to review the new evidence to understand whether the robotic approach could find some clear indication also in left colectomy.

**Methods:**

A systematic review of studies published from 2004 to 2022 in the Web of Science, PubMed, and Scopus databases and comparing laparoscopic (LLC) and robotic left colectomy (RLC) was performed. All comparative studies evaluating robotic left colectomy (RLC) versus laparoscopic (LLC) left colectomy with at least 20 patients in the robotic arm were included. Abstract, editorials, and reviews were excluded. The Newcastle–Ottawa Scale for cohort studies was used to assess the methodological quality. The random-effect model was used to calculate pooled effect estimates.

**Results:**

Among the 139 articles identified, 11 were eligible, with a total of 52,589 patients (RLC, *n* = 13,506 versus LLC, *n* = 39,083). The rate of conversion to open surgery was lower for robotic procedures (RR 0.5, 0.5–0.6; *p* < 0.001). Operative time was longer for the robotic procedures in the pooled analysis (WMD 39.1, 17.3–60.9, *p* = 0.002). Overall complications (RR 0.9, 0.8–0.9, *p* < 0.001), anastomotic leaks (RR 0.7, 0.7–0.8; *p* < 0.001), and superficial wound infection (RR 3.1, 2.8–3.4; *p* < 0.001) were less common after RLC. There were no significant differences in mortality (RR 1.1; 0.8–1.6, *p* = 0.124). There were no differences between RLC and LLC with regards to postoperative variables in the subgroup analysis on malignancies.

**Conclusions:**

Robotic left colectomy requires less conversion to open surgery than the standard laparoscopic approach. Postoperative morbidity rates seemed to be lower during RLC, but this was not confirmed in the procedures performed for malignancies.

**Supplementary information:**

The online version contains supplementary material available at 10.1007/s00384-022-04194-8.

## Introduction

Since its introduction, robotic surgery has been an increasingly real alternative to laparoscopy in colorectal surgery. Its use was shown to be associated with lower conversion rates, improved functional outcomes, and an increased number of lymph node harvested both in right colectomy and in rectal resection [1–5].

Those results may be due to the intrinsic characteristics of the robotic platform (magnified 3-dimensional visualization, stable platform, and seven degrees of freedom wrist) which allow precise and delicate dissection along with a simplified way of intra-corporeal suturing.

However, those advantages may not be clearly evident in left colectomies, where the surgeon operates in an “open field” and does not routinely require intraoperative suturing.

Still, few authors reported advantages also in performing this procedure: Some showed that robotic left colectomy (RLC) may be associated with lower morbidity and conversion rates along with a shorter hospital stay [6–10], while others found that the robotic platform offered minor advantages for this procedure [11–16].

The last meta-analysis performed on the topic was published in 2016 by Lorenzon et al. who did not find any differences between the two procedures [17]. However, at that time, pooled outcomes may have been biased by the fact that the meta-analyses included only seven studies for a total of 143 patients.

Since then, several studies [6–8, 10–13, 16, 18–28] have been conducted on the comparison between robotic and laparoscopic left colectomy. For this reason, this study aimed to review the new evidence to understand whether this approach could find some clear indication also in left colectomy.

## Methods

### Literature search strategy

This study was performed and reported according to the 2010 Preferred Reporting Items for Systematic Reviews and Meta-Analyses (PRISMA) [29] and AMSTAR 2 guidelines [30]. A systematic literature search was performed in Web of Science, PubMed, and Scopus databases for pertinent studies published between November 2004 and March 2022. Search terms used were *("robot"[All Fields] OR "robot s"[All Fields] OR "robotically"[All Fields] OR "robotics"[MeSH Terms] OR "robotics"[All Fields] OR "robotic"[All Fields] OR "robotization"[All Fields] OR "robotized"[All Fields] OR "robots"[All Fields]) AND "left"[All Fields] AND ("colectomy"[MeSH Terms] OR "colectomy"[All Fields] OR "colectomies"[All Fields])*. “Related articles” function and manual reference screening were also used.

Results from the databases were assessed to create a single list of articles for screening. Titles, abstracts and, subsequently, full-text articles were checked and selected independently by two authors (AB and LS). Disagreement on eligibility was addressed by discussion and followed by consensus.

Grey literature search was not performed.

### Eligibility criteria

Only full-text studies in English language which compared RLC versus LLC were included. Comparative studies with less than 20 patients per arm and on pediatric patients were excluded. Abstract, editorials, and reviews were also excluded from the analysis at this point of study selection.

Primary endpoint was conversion to open surgery. Secondary endpoints were blood loss, postoperative morbidity and mortality, harvested lymph nodes, anastomotic leak, postoperative hemorrhage, abdominal abscess, postoperative ileus, time to first flatus, non-surgical complications, wound infections, hospital stay, and incisional hernia and costs.

For overlapping series, only the most recent study was included.

### Assessment of methodological quality and data extraction

Methodological quality was evaluated independently by two authors (AB and LS). The Newcastle–Ottawa Scale [31] for cohort studies which assesses the methodological quality based on quality of selection, comparability, and outcome of study participants was used in this review.

Data extracted included study characteristics (country of origin, study period, study design), patients’ characteristics [age, sex, and body mass index (BMI), indication for surgery, American Society of Anesthesiologists (ASA) score], and intraoperative (type of robotic platform used, type of anastomosis, operative time, blood loss, conversion to open surgery, and harvested lymph nodes) and postoperative variables (in-hospital mortality, overall morbidity, Clavien-Dindo morbidity [32] anastomotic leak, postoperative hemorrhage, postoperative ileus, time to first flatus, and hospital stay).

### Subgroup analysis

A subgroup analysis was performed on those series presenting left colectomies performed for malignancy.

### Statistical analysis

Categorical variables were presented as the weighted pooled rates with 95% confidence intervals (95% CI) exploiting by the Freeman–Tukey transformation [33], and their comparisons were shown as relative risk (RR). Continuous variables were presented in weighted pooled means and 95% CI using the inverse variance method. Comparisons were reported as pooled weighted mean difference (WMD). When included studies presented continuous variables as median and interquartile range (or median and range), they were converted in mean and standard deviation (SD) as recommended by Hozo et al. [34]. Heterogeneity between included studies was explored by inconsistency (*I*^2^) statistics [35]. Funnel plots were constructed to evaluate the risk of publication bias. When outliers were present, a sensitivity analysis was performed excluding those studies. Statistical analysis was performed using MedCalc Statistical Software version 15.8 (MedCalc Software bvba, Ostend, Belgium; https://www.medcalc.org; 2015) and StataCorp 2017 (Stata Statistical Software: Release 15, StataCorp LLC).

## Results

**Fig. 1:**
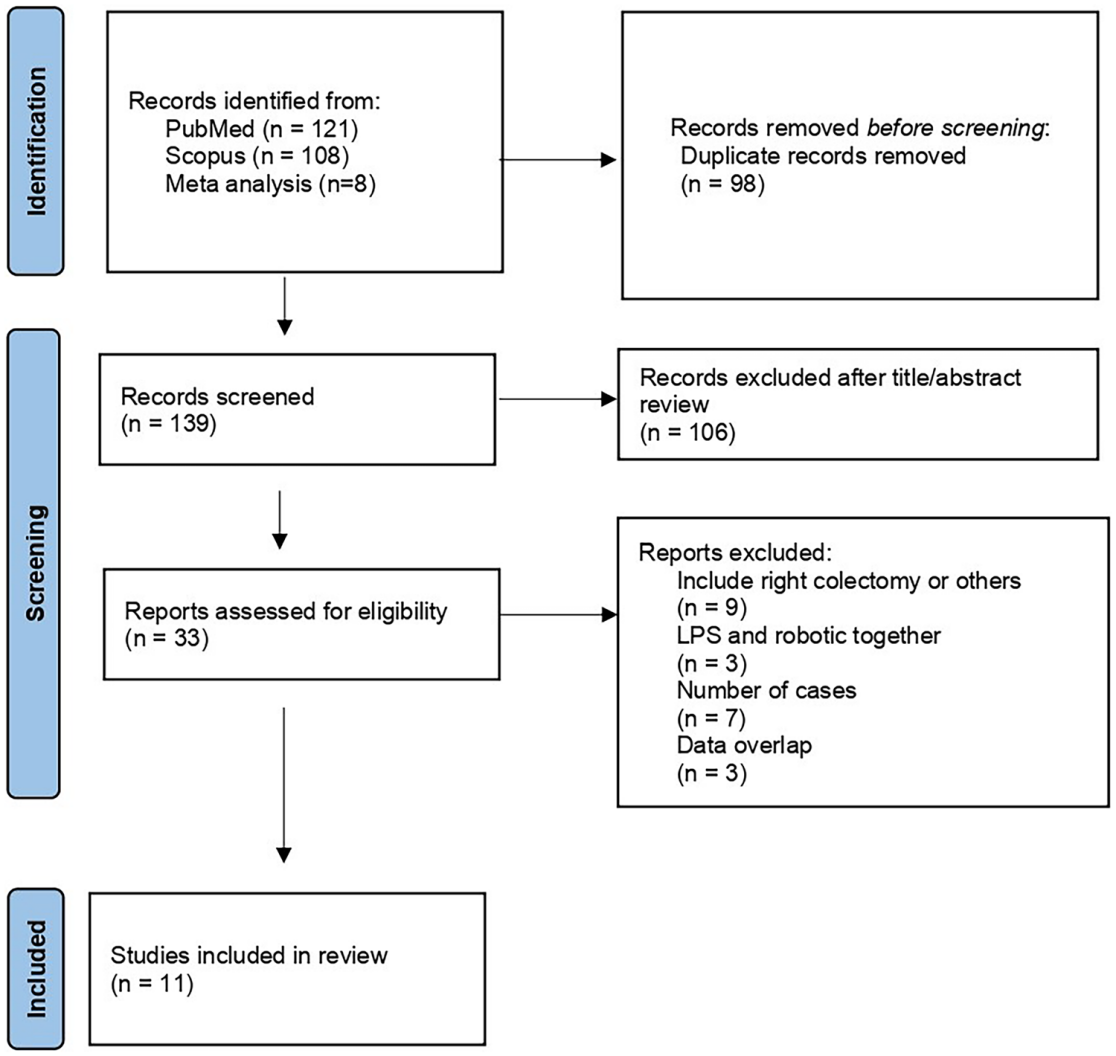
PRISMA flow chart

Database search and manual screening of reference lists yielded a total of 139 potentially relevant articles (Fig. 1) after excluding duplicates. Twenty-one studies were excluded [18–28, 36–46]. The reasons for exclusion were cohort with less than 20 RLC cases [25, 41–46], impossibility to extract data on RLC [18–24, 36–40] or data overlap [26–28]. Eleven studies published between 2013 and 2022 were considered eligible for data extraction and were therefore included in the meta-analysis [6–16]. A total of 52,589 individual patients who underwent RLC (*n* = 13,506) or LLC (*n* = 39,083) were identified. The quality assessments for each study are shown in Supplementary Table 1.

### Study and patient characteristics

Details of included study are shown in Table 1. In two studies, the analyses of outcomes were performed exploiting large multi-institutional databases [6, 10]. Indications for performing minimally invasive left colectomies were diverticular disease [6, 7, 10, 12, 14, 16], complicated diverticular disease [8, 9] or malignancy [7, 10, 11, 13–15]. All but three studies [6, 10, 14] reported the type and version of the robotic platform used.

**Table 1:** Study characteristics and quality assessment

First author	Country	Study period	Study design	Robot	Laparoscopic	Robotic	Q.A	Monocentric
Lim	Korea	2006–2008	Retrospective cohort	Si	146	34	8	Yes
Casillas	USA	2005–2012	Prospective cohort, PSM		82	68	6	Yes
Cassini	Italy	2009–2017	Retrospective cohort	S	92	64	8	Yes
Maciel	USA	2009–2013	Retrospective cohort	Da Vinci	55	20	7	Yes
Bilgin	Turkey	2011–2018	Retrospective cohort	Xi	22	20	8	Yes
Al Temimi	USA	2012–2014	Retrospective cohort, PSM		439	439	7	Database
Kim	Korea	2012–2017	Retrospective cohort	Si/Xi	51	20	8	Yes
Xu	China	2012–2018	Retrospective cohort	Si	255	205	8	Yes
Beltzer	Germany	2013–2018	Case control retrospective	Xi	46	60	8	Yes
Mlambo	USA	2013–2019	Retrospective cohort		37,543	12,400	8	Database
Gass	Swiss	2015–2019	Retrospective cohort, PSM	Si/Xi	352	176	8	Yes

*QA* quality assessment, *PSM* propensity score matched

Pooled patients’ characteristics are reported in Table 2. Preoperative variables (age, BMI, ASA score, and sex) did not differ between the two groups (*p* > 0.05). Seven studies [8, 9, 11, 12, 14–16] reported the technique adopted to perform the colorectal anastomosis which was performed in all studies after undocking.

**Table 2:** Pooled patients' characteristics of robotic versus laparoscopic left colectomies

Variable	No. Of patients	Robotic	Laparoscopic	*P*	*I*^2^ (95% CI)	References
Age (years)	52,589	60.1 (57.2–63.0)	60.5 (58.2–62.9)	0.223	0.0 (0.0–61.3)	6–16
Sex, m	52,589	58.6 (43.4–73.1)	50.9 (45.9–55.8)	0.289	89.2 (82.8–93.3)	6–16
BMI	2496	26.8 (25.7–27.9	26.2 (25.1–27.4)	1.00	0.0 (0.0–0.0)	6–9, 11–13, 15, 16
ASA > 2 (%)	2043	16.0 (7.9–26.2)	14.6 (6.8–24.6)	0.261	0.0 (0.0–43.2)	6, 8, 11–16
Benign disease (%)	51,878	70.4 (44.8–90.6)	67.4 (36.0–91.9)	0.228	0.0 (0.0–0.0)	6–10, 12, 14, 16

*I*^2^ < 25% were interpreted as low heterogeneity, 25 ≤ *I*^2^ ≤ 50% as medium, between 50 < *I*^2^ ≤ 75% as substantial, *I*^2^ > 75% as considerable

Three studies [8, 9, 11] reported that RLC was performed with three robotic arms, while four studies included procedures with four arms [12, 13, 15, 16].

### Perioperative outcomes

#### Operative time

**Fig. 2:**
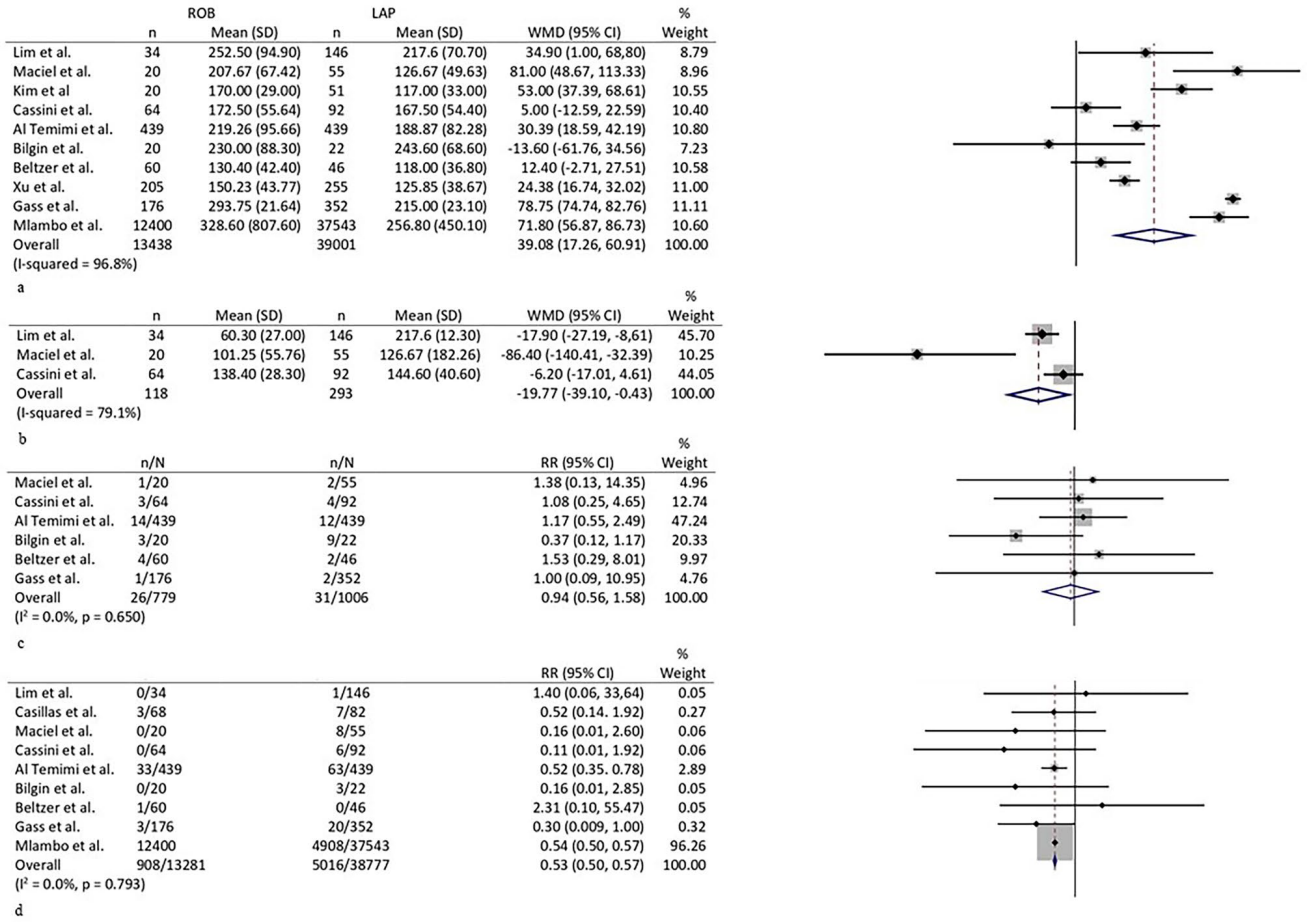
Forest plots of **a** operative time, **b** blood loss, **c** stoma, and **d** conversion to open surgery

Ten studies reported details on operative time [6–13, 15, 16]. The pooled mean was higher in the robotic group (215 min, 163–267 versus 177, 144–210; *p* = 0.002; WMD 39.1, 17.3–60.9) (Fig. 2a). Heterogeneity among studies was high (*I*^2^ 96.8%, 96.2–98.9; *p* < 0.001). A sensitivity analysis excluding the three outliers [9, 11, 13] on the funnel plot confirmed longer operative times in the robotic group (WMD 33.6 min, 5.3–61.9; *p* = 0.006; *I*^2^ 17.3%, 0.0–83.8; *p* = 0.305).

#### Blood loss

This variable was reported by three studies [8, 9, 15]. The WMD favored the robotic group (WM −19.8 ml, −39.1 to −0.4; *p* = 0.050) (Fig. 2b). High heterogeneity was found among studies (*I*^2^ 79.1%, 55.9–94.9).

#### Stoma

Data on this variable were present in six studies [6–9, 16]. RR was 0.9 (0.6–1.6; *p* = 0.828) (Fig. 2c). Low heterogeneity was present among studies (*I*^2^ 0.0%, 0.0–62.9; *p* = 0.650), and no obvious evidence of bias were seen in the funnel plot.

#### Conversion to open surgery

All studies reported details about conversion to open surgery [6–16]. Two studies [11, 13] registered no conversions in both groups. The pooled rate of conversion was 2.2% (0.6–4.7) in the robotic group versus 6.1 (2.4–11.2) in the laparoscopic group (*p* < 0.001), and RR was 0.5 (0.5–0.6) (Fig. 2d). Heterogeneity among studies was low (*I*^2^ 0.0%, 0.0–55.2; *p* = 0.793). No evidence of significant bias was seen in the funnel plot.

#### Postoperative complications

**Fig. 3:**
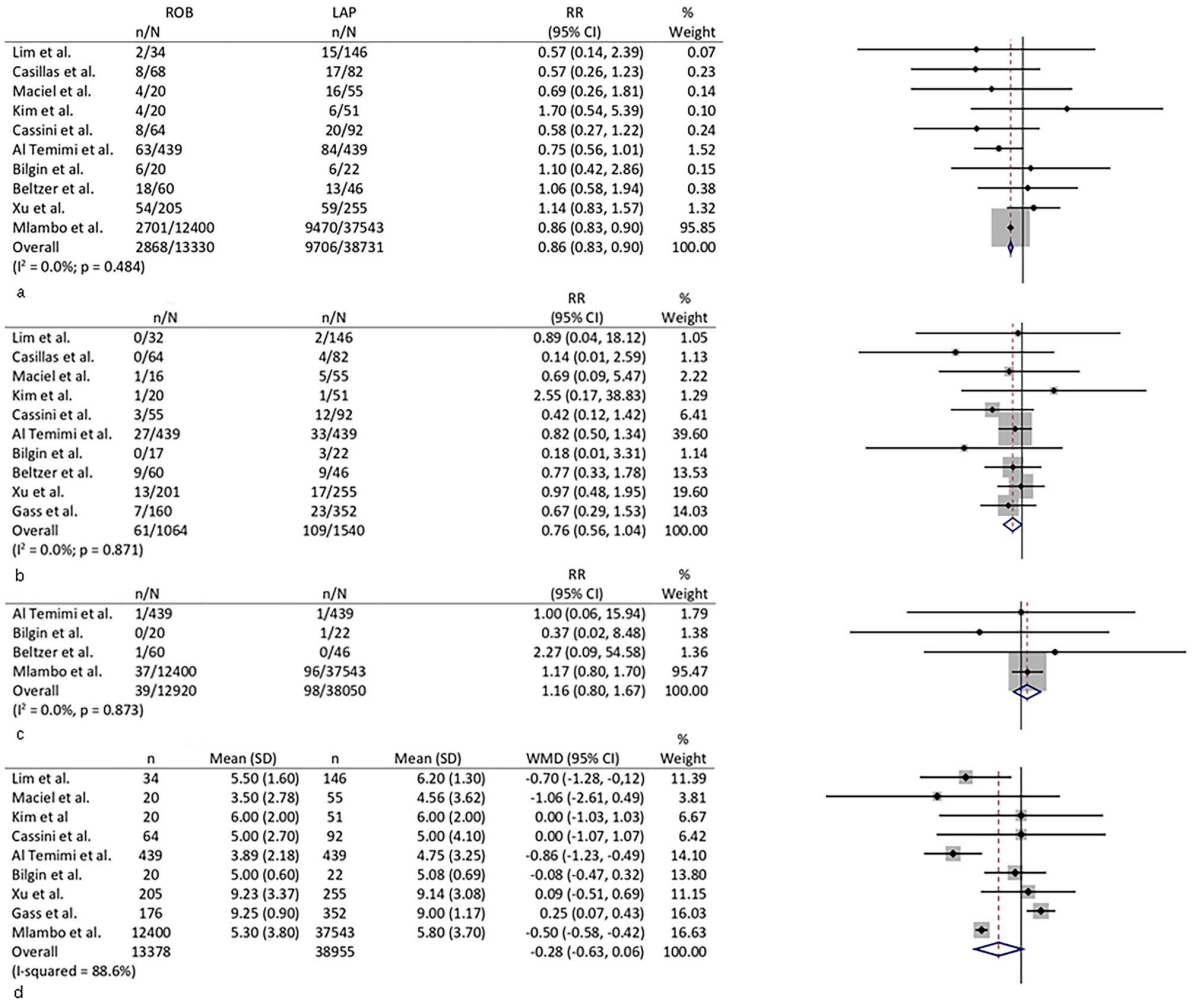
Forest plots of **a** overall complication, **b** Clavien-Dindo > 2 complications, **c** mortality, and **d** hospital stay

In ten studies, data on overall postoperative complications could be extracted [6, 8–16]. The pooled RR showed higher risk of complication after LLC (RR 0.9, 0.8–0.9, *p* < 0.001) (Fig. 3a). *I*^2^ was 0.0% (0.0–60.4).

Ten studies reported complications using the Clavien-Dindo scale [6–9, 11–16]. The RR was 0.8 (0.6–1.0; *p* = 0.055) (Fig. 3b). Heterogeneity between studies was low (*I*^2^ 0.0, 0.0–39.9; *p* = 0.778). The funnel plots for both variables did not show any obvious publication bias.

#### Postoperative mortality

All but one study [7] reported the postoperative mortality. No mortality was reported in both groups in six studies [8, 9, 11, 13–15]. No differences were found between RLC and LLC with regard to this variable (RR 1.2, 0.8–1.7; *p* = 0.477). *I*^2^ was 0.0 (0.0–87.1) (Fig. 3c). The funnel plot did not show evidence of significant bias among studies.

#### Hospital stay

Nine studies reported details about length of hospital stay [6–13, 15]. No differences were seen between the two groups according to this variable with a WMD of −0.3 (−0.6–0.1; *p* = 0.120). Heterogeneity was high between the studies (*I*^2^ 88.6%, 70.2–97.4; *p* < 0.001) (Fig. 3d), and funnel plot showed two outliers [6, 7]. The sensitivity analysis excluding these two studies showed a weighted mean difference in favor of the robotic group with a WMD of −0.3 (−0.6 to −0.1) (*I*^2^ 27.5%, 0.0–68.7; *p* = 0.219).

#### Postoperative bleeding

**Fig. 4:**
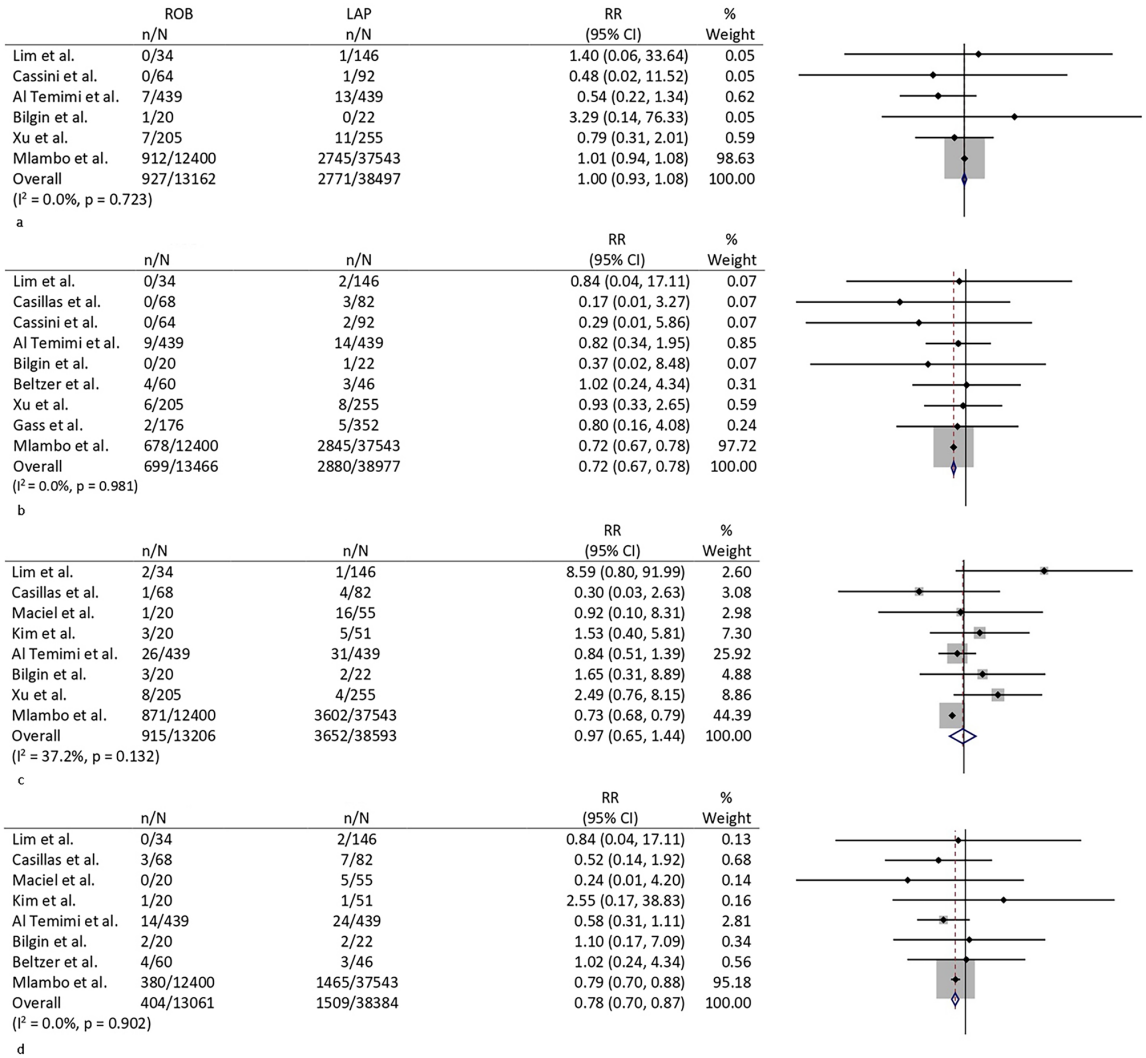
Forest plots of **a** postoperative bleeding, **b** anastomotic leak, **c** ileus, and **d** superficial wound infection

A total of six studies [6, 8, 10, 12, 13, 15] reported the variables. No differences were seen between the groups with a pooled RR of 1.0 (0.9–1.1; *p* = 0.989) (Fig. 4a). Low heterogeneity was found between the studies (*I*^2^ 0.0, 0.0–56.7; *p* = 0.723). The funnel plot did not show evidence of significant bias among studies.

#### Anastomotic leak

Data on anastomotic leak could be extracted in ten studies [6–8, 10–16]. Kim et al. reported no leaks in both groups [11]. The RR was 0.7 (0.7–0.8; *p* < 0.001) in favor of RLC (Fig. 4b). Heterogeneity was not present (*I*^2^ 0.0, 0.0–0.0, *p* = 0.981). The funnel plot showed a nearly symmetrical distribution.

#### Postoperative ileus

A total of 8 studies reported data on postoperative ileus [6, 9–15]. The RR was 0.97 (0.65–1.44; *p* = 0.876). *I*^2^ was 37.2 (0.0–72.3; *p* = 0.132) (Fig. 4c). The funnel plot did not show any obvious publication bias.

#### Superficial wound infection

Pooled analysis could be performed on eight studies [6, 9–12, 14–16]. The pooled rate of superficial wound infection was higher in the laparoscopic group (4.9%, 3.4–6.8 versus 3.1, 2.8–3.4; *p* < 0.001) (Fig. 4d). Heterogeneity among studies was low with an *I*^2^ of 0.0% (0.0–20.0; *p* = 0.902). No clear signs of publication bias were seen in the funnel plot.

#### Malignant disease subgroup analysis

Three studies [11, 13, 15] were included in this subgroup analysis. Pooled perioperative variables are shown in Table 3. As indicated, data were only from two out of three studies only for anastomotic leak [13, 15] and resection margin [11, 15]. Operative time was longer with the robotic approach (WMD 30.1, 23.4–36.8; *p* = 0.002). No significant differences were found in any postoperative outcome variables. The median number of lymph nodes harvested (WMD −0.3, −1.2–0.5; *p* = 0.344) [11, 13, 15] and the measure of the closest margin (WMD 0.1, −1.1–1.2; *p* = 0.870) [11, 15] were similar between the groups. The funnel plots did not show evidence of significant bias among studies.

**Table 3:** Pooled analyses of robotic versus laparoscopic left colectomies performed for malignancy

Variable	No. of patients	Robotic	Laparoscopic	*P*	*I*^2^ (95% CI)	References
Age (years)	711	60.1 (58.7–61.3)	59.3 (57.4–61.2)	0.872	0.0 (0.0–81.3)	11, 13, 15
Sex –m-	711	60.9 (54.9–66.7)	64.1 (59.6–68.4)	0.316	11.8 (0.0–97.0)	11, 13, 15
BMI	711	24.8 (24.6–25.2)	24.2 (23.5–24.9)	0.203	49.4 (0.0–85.3)	11, 13, 15
ASA > 2	711	10.7 (7.3–15.1)	7.4 (5.2–11.0)	0.324	0.0 (0.0–91.6)	11, 13, 15
Stage III.IV tumors	711	30.4 (24.6–36.5)	36.3 (32.0–40.8)	0.156	32.7 (0.0–97.6)	11, 13, 15
Operative time (min)	711	186 (150–222)	153 (103–203)	0.002	84.2 (52.6–94.7)	11, 13, 15
Conversion to open surgery	711	0.2 (0.0–1.2)	0.3 (0.0–1.1)	n.a		11, 13, 15
Complications	711	18.0 (6.7–33.2)	14.4 (7.4–25.4)	0.477	0.0 (0.0–95.1)	11, 13, 15
Complications Clavien-Dindo ≥ 3	711	5.5 (3.0–8.9)	3.6 (0.9–8.1)	0.997	0.0 (0.0–86.2)	11, 13, 15
Anastomotic leak	711	2.5 (1.0–5.3)	2.1 (0.8–4.1)	0.873	0.0 (0.0–0.0)	13, 15
Time to first flatus	711	2.6 (1.5–3.8)	2.7 (1.8–3.7)	0.255	26.9 (0.0–97.5)	11, 13, 15
Lymph nodes harvested	711	15.7 (11.7–19.5)	17.5 (13.3–21.7)	0.304	50.4 (0.0–85.7)	11, 13, 15
Margin (cm)	251	7.0 (3.5–10.5)	8.1 (1.7–14.6)	0.870	67.8 (0.0–92.7)	11, 15
Mortality	711	0.9 (0.1–5.0)	0.2 (0.0–1.4)	n.a		11, 13, 15
Hospital stay (days)	711	6.9 (4.4–9.4)	7.1 (5.1–9.1)	0.407	69.2 (0.0–91.0)	11, 13, 15

*I*^2^ < 25% were interpreted as low heterogeneity, 25 ≤ *I*^2^ ≤ 50% as medium, between 50 < *I*^2^ ≤ 75% as substantial, *I*.^2^ > 75% as considerable. *n.a*. not available as not enough events

## Discussion

The use of the robotic platform to perform left colectomy was associated with improved intraoperative and postoperative outcomes.

The rate of conversion to open surgery was lower in the robotic group, and this is consistent with the data from the literature on robotic surgery in basically all surgical fields [1, 5, 47]. Giuliani et al. [48] in their meta-analyses on left colonic diverticular disease found a pooled odds ratio of 0.56 (*p* < 0.001) for conversions with the robotic approach.

Our sub-analysis on left colectomies (259 RLC versus 452 LLC) performed for malignant disease did not find a difference between the groups with regard to conversion to open surgery as they occurred only in one patient in the laparoscopic group. This may be due to the fact that the three studies in this sub-analysis included a small rate of patients with T4 tumors. Still, we could speculate that, in particular settings such as advanced stages, the robotic platform with its magnified 3-dimensional visualization, stable platform, and seven degrees of freedom wrist might allow a more precise and delicate dissection guaranteeing lower rates of conversion to open surgery. Comparative prospective studies on T4 tumors are warranted to confirm this hypothesis.

Postoperative complications were more common after LLC. However, it is important to note that this result is mostly owing to data from the two largest studies, both of which used multi-institutional national databases [6, 10]. Those studies did not report several variables which may have influenced the postoperative complications. In addition, this outcome might have also been linked to the higher rate of anastomotic leak which apparently favored the robotic approach. In light of this, it must be noted that six studies [8, 9, 12, 14–16] reported that colorectal anastomosis was routinely performed with standard laparoscopy also in the robotic arms.

Again, this difference was not seen in the sub-analysis on oncologic patients, and this may support the hypothesis that a selection bias may have been present, since patients at higher risk or with more heterogeneous indications (e.g., complicated diverticular disease) were included in the laparoscopic groups.

In our opinion, those results should highlight the non-inferiority of the robotic approach rather its superiority in terms of safety.

As expected, operative time was longer in the robotic group. This outcome is extremely common in the surgical literature comparing robotic and laparoscopic procedures [46, 48]. First, this may be partly due to the docking time. Second, we did not have any details on learning curve which might have influenced the operative time of the robotic group. Finally, as it has already been highlighted elsewhere [49], the higher dexterity and the extremely accurate view of the operating field provided by the robotic platform may lead to such a meticulous dissection of tissues which may be more commonly seen in microsurgery rather than laparoscopy; this might result in a procedure requiring a prolonged operative time.

Given its safety parameters and the wide operative field required for the procedure, we believe, as it has already been reported by others [7], that the robotic approach may be of use in left colectomy for training purposes. In this case, the surgical fellow may familiarize with the robotic platform in a user-friendly surgical field which may be preparatory for robotic rectal anterior resection. Expert surgeon might benefit from the robotic approach during left colectomy in “difficult cases” such as T4 tumors or complicated diverticular disease during which the technical characteristics of the platform may be extremely useful.

As only two studies, representing the 0.8% of the pooled cohort, reported long-term outcomes of oncologic patients [13, 15], we chose not to analyze survival variables in the pooled analysis. In both studies, the overall survival and disease-free survival were similar between RLC and LLC. Recently, the long-term outcomes of a randomized controlled trial on robotic versus laparoscopic right colectomy has been published [50]: the authors found that the combined 5-year overall survival rates for all stages were 91.1% in the robotic group versus 91.0% in the laparoscopic one (*p* = 0.678). These results suggest that the robotic approach should not affect the long-term outcomes of oncologic patients; however, given the low number of studies on robotic surgery reporting data on survival, future studies on the topic should consider the inclusions of these variables in their analysis.

To date, no randomized controlled trials (RCTs) were performed on the comparison between RLC and LLC. In the field of robotic colorectal surgery, it was possible to produce level 1a evidence only for rectal resection. Li et al. [51] performed a meta-analysis including the RCTs on robotic versus laparoscopic rectal surgery for rectal cancer. The authors found results similar to the present meta-analysis, showing lower pooled conversion rate in the robotic group (odds ratio 0.3, 0.1–1.0; *p* = 0.04) and comparable postoperative outcomes. These data may corroborate our findings for left colectomy, but randomized prospective trials are needed to confirm the potential benefits of using the robotic platform.

A consideration of surgeons’ comfort should be mentioned in the analysis of the comparison between robotic and laparoscopic surgery. The seated position with the arm and forehead support, typical of the robotic platform, has been claimed to represent a more comfortable and less physically demanding work posture. In 2017, the finding of a systematic review based on 15 studies suggested that robotic-assisted laparoscopy may be less strenuous compared with conventional laparoscopy [52]. Recently, Dalager et al. [53] analyzed the posture and muscle strain in 12 surgeons during colorectal procedures with the two approaches, and they found that the robotic approach was less demanding on posture.

These findings, which might be currently considered a side benefit of the robotic platform, may contribute to the widespread adoption of this approach in colorectal surgery in the future.

This meta-analysis carries a few limitations linked to the biases of the included studies. The two studies with the largest cohorts [6, 10] deriving from massive national database may include very heterogeneous groups of patients for whom it is difficult to extract important details influencing the outcomes. In light of this, it may be recommended to be aware of a possible selection bias when interpreting these results as those studies have a non-negligible impact in addressing the pooled outcomes. Finally, the analyses on costs could not be performed. The three studies [10, 11, 13] which reported this outcome and favored the laparoscopic approach reported heterogeneous variables and definitions which cannot be pooled. Still, it is desirable that the economic aspects on the use of the robotic technology should be assessed in future studies in order to highlight the financial sustainability of each proposed procedure.

In conclusion, robotic left colectomy requires less conversion to open surgery than the standard laparoscopic approach. Postoperative morbidity rates seemed to be lower during RLC; however, this may be due to selection bias. More studies reporting prospective homogenous cohort are warranted to highlight possible advantages in using the robotic platform for left colectomy.

## Supplementary Information

Below is the link to the electronic supplementary material.

Supplementary file1 (DOCX 26 KB)

## References

[1] Solaini L, Bazzocchi F, Cavaliere D, Avanzolini A, Cucchetti A, Ercolani G (2018). Robotic versus laparoscopic right colectomy: an updated systematic review and meta-analysis. Surg Endosc.

[2] Waters PS, Cheung FP, Peacock O, Heriot AG, Warrier SK, O’Riordain DS (2020). Successful patient-oriented surgical outcomes in robotic *vs* laparoscopic right hemicolectomy for cancer – a systematic review. Colorectal Dis.

[3] Milone M, Manigrasso M, Velotti N, Torino S, Vozza A, Sarnelli G (2019). Completeness of total mesorectum excision of laparoscopic versus robotic surgery: a review with a meta-analysis. Int J Colorectal Dis.

[4] Grass JK, Chen C, Melling N, Lingala B, Kemper M, Scognamiglio P (2021). Robotic rectal resection preserves anorectal function: systematic review and meta-analysis. The International Journal of Medical Robotics and Computer Assisted Surgery.

[5] Solaini L, Perna F, Cavaliere D, Vaccaro C, Avanzolini A, Cucchetti A (2021). Average treatment effect of robotic versus laparoscopic rectal surgery for rectal cancer. Int J Med Robot Comp Assiste Surg.

[6] Al-Temimi MH, Chandrasekaran B, Agapian J, Peters WR, Wells KO (2019). Robotic versus laparoscopic elective colectomy for left side diverticulitis: a propensity score–matched analysis of the NSQIP database. Int J Colorectal Dis.

[7] Gass JM, Daume D, Schneider R, Steinemann D, Mongelli F, Scheiwiller A (2022). Laparoscopic versus robotic-assisted, left-sided colectomies: intra- and postoperative outcomes of 683 patients. Surg Endosc.

[8] Cassini D, Depalma N, Grieco M, Cirocchi R, Manoochehri F, Baldazzi G (2019). Robotic pelvic dissection as surgical treatment of complicated diverticulitis in elective settings: a comparative study with fully laparoscopic procedure. Surg Endosc.

[9] Maciel V, Lujan HJ, Plasencia G, Zeichen M, Mata W, Jorge I (2014). Diverticular disease complicated with colovesical fistula: laparoscopic versus robotic management. Int Surg.

[10] Mlambo B, Shih IF, Li Y, Wren SM (2022). The impact of operative approach on postoperative outcomes and healthcare utilization after colectomy. Surgery.

[11] Kim JC, Lee JL, Yoon YS, Kim CW, Park IJ, Lim SB (2018). Robotic left colectomy with complete mesocolectomy for splenic flexure and descending colon cancer, compared with a laparoscopic procedure. Int J Med Robot Comp Assist Surg.

[12] Bilgin IA, Bas M, Benlice C, Esen E, Ozben V, Aytac E (2020). Totally laparoscopic and totally robotic surgery in patients with left-sided colonic diverticulitis. Int J Med Robot Comp Assist Surg.

[13] Xu M, Zhao Z, Jia B, Liu R, Liu H (2021). Perioperative and long-term outcomes of robot-assisted versus laparoscopy-assisted hemicolectomy for left-sided colon cancers: a retrospective study. Updat Surg.

[14] Casillas MA, Leichtle SW, Wahl WL, Lampman RM, Welch KB, Wellock T (2014). Improved perioperative and short-term outcomes of robotic versus conventional laparoscopic colorectal operations. Am J Surg.

[15] Lim DR, Min BS, Kim MS, Alasari S, Kim G, Hur H (2013). Robotic versus laparoscopic anterior resection of sigmoid colon cancer: comparative study of long-term oncologic outcomes. Surg Endosc.

[16] Beltzer C, Knoerzer L, Bachmann R, Axt S, Dippel H, Schmidt R (2019). Robotic versus laparoscopic sigmoid resection for diverticular disease: a single-center experience of 106 cases. J Laparoendosc Adv Surg Tech.

[17] Lorenzon L, Bini F, Balducci G, Ferri M, Salvi PF, Marinozzi F (2016). Laparoscopic versus robotic-assisted colectomy and rectal resection: a systematic review and meta-analysis. Int J Colorectal Dis.

[18] Nolan HR, Smith BE, Honaker MD (2018). Operative time and length of stay is similar between robotic assisted and laparoscopic colon and rectal resections. J Robot Surg.

[19] Ngu JC, Teo N (2022) Robotic assistance is technically superior to conventional laparoscopy in hemicolectomies. Int J Med Robot Comp Assist Surg e2367. 10.1002/rcs.2367 10.1002/rcs.2367 35015929

[20] Diaz SE, Lee YF, Bastawrous AL, Shih IF, Lee SH, Li Y et al (2022) Comparison of health-care utilization and expenditures for minimally invasive vs. open colectomy for benign disease. Surg Endoscop. 10.1007/s00464-022-09097-x 10.1007/s00464-022-09097-x PMC9485164 35194661

[21] Nasseri Y, Kasheri E, Oka K, Cox B, Cohen J, Ellenhorn J (2021). Minimally invasive right versus left colectomy for cancer: does robotic surgery mitigate differences in short-term outcomes?. J Robot Surg.

[22] Chang TC, Lin EK, Lu YJ, Huang MT, Chen CH (2021). Single-incision robotic colectomy versus single-incision laparoscopic colectomy: A matched case control study. Asian J Surg.

[23] Wei D, Johnston S, Goldstein L, Nagle D (2020). Minimally invasive colectomy is associated with reduced risk of anastomotic leak and other major perioperative complications and reduced hospital resource utilization as compared with open surgery: a retrospective population-based study of comparative effectiveness and trends of surgical approach. Surg Endosc.

[24] Vasudevan V, Reusche R, Wallace H, Kaza S (2016). Clinical outcomes and cost–benefit analysis comparing laparoscopic and robotic colorectal surgeries. Surg Endosc.

[25] Palomba G, Dinuzzi VP, Capuano M, Anoldo P, Milone M, De Palma GD (2021). Robotic versus laparoscopic colorectal surgery in elderly patients in terms of recovery time: a monocentric experience. J Robot Surg.

[26] Dolejs SC, Waters JA, Ceppa EP, Zarzaur BL (2017). Laparoscopic versus robotic colectomy: a national surgical quality improvement project analysis. Surg Endosc.

[27] Bastawrous AL, Landmann RG, Liu Y, Liu E, Cleary RK (2020). Incidence, associated risk factors, and impact of conversion to laparotomy in elective minimally invasive sigmoidectomy for diverticular disease. Surg Endosc.

[28] Ogilvie JW, Saunders RN, Parker J, Luchtefeld MA (2019). Sigmoidectomy for diverticulitis—a propensity-matched comparison of minimally invasive approaches. J Surg Res.

[29] Moher D, Liberati A, Tetzlaff J, Altman DG (2009). Preferred reporting items for systematic reviews and meta-analyses: The PRISMA statement. Int J Surg.

[30] Shea BJ, Bouter LM, Peterson J, Boers M, Andersson N, Ortiz Z (2007). External validation of a measurement tool to assess systematic reviews (AMSTAR). PLoS ONE.

[31] Lo CKL, Mertz D, Loeb M (2014). Newcastle-Ottawa scale: comparing reviewers’ to authors’ assessments. BMC Med Res Methodol.

[32] Dindo D, Demartines N, Clavien PA (2004). Classification of surgical complications: a new proposal with evaluation in a cohort of 6336 patients and results of a survey. Ann Surg.

[33] Freeman MF, Tukey JW (1950). Transformations related to the angular and the square root. Ann Math Stat.

[34] Hozo SP, Djulbegovic B, Hozo I (2005). Estimating the mean and variance from the median, range, and the size of a sample. BMC Med Res Methodol.

[35] Higgins JPT, Thompson SG, Deeks JJ, Altman DG (2003). Measuring inconsistency in meta-analyses. BMJ.

[36] Spinoglio G, Summa M, Priora F, Quarati R, Testa S (2008). Robotic colorectal surgery: first 50 cases experience. Dis Colon Rectum.

[37] D’Annibale A, Morpurgo E, Fiscon V, Trevisan P, Sovernigo G, Orsini C (2004). Robotic and laparoscopic surgery for treatment of colorectal diseases. Dis Colon Rectum.

[38] Shin JY (2012). Comparison of short-term surgical outcomes between a robotic colectomy and a laparoscopic colectomy during early experience. Journal of the Korean Society of Coloproctology.

[39] Deutsch GB, Sathyanarayana SA, Gunabushanam V, Mishra N, Rubach E, Zemon H et al (2012) Robotic vs. laparoscopic colorectal surgery: an institutional experience. Surg Endoscop, 26(4):956–963. 10.1007/s00464-011-1977-6 10.1007/s00464-011-1977-6 22044968

[40] Davis BR, Yoo AC, Moore M, Gunnarsson C (2014). Robotic-assisted versus laparoscopic colectomy: cost and clinical outcomes. J Soc Laparoscop Robot Surg.

[41] Guerrieri M, Campagnacci R, Sperti P, Belfiorigesuita, R., Ghiselli, R.  G (2015). Totally robotic *vs* 3D laparoscopic colectomy: a single centers preliminary experience. World J Gastroenterol.

[42] Ferrara F, Piagnerelli R, Scheiterle M, Di Mare G, Gnoni P, Marrelli D, Roviello F (2015). Laparoscopy versus robotic surgery for cancer: a single-center initial experience. Surg Innov.

[43] Rawlings AL, Woodland JH, Vegunta RK, Crawford DL (2007). Robotic versus laparoscopic colectomy. Surg Endosc.

[44] Woeste G, Bechstein WO, Wullstein C (2005). Does telerobotic assistance improve laparoscopic colorectal surgery?. Int J Colorectal Dis.

[45] Delaney CP, Lynch CA, Senagore AJ, Fazio VW (2003). Comparison of robotically performed and traditional laparoscopic colorectal surgery. Dis Colon Rectum.

[46] Elliott PA, McLemore EC, Abbass MA, Abbas MA (2015). Robotic versus laparoscopic resection for sigmoid diverticulitis with fistula. J Robot Surg.

[47] Cavaliere D, Solaini L, Di Pietrantonio D, D’Acapito F, Tauceri F, Framarini M (2018). Robotic vs laparoscopic splenectomy for splenomegaly: a retrospective comparative cohort study. Int J Surg.

[48] Giuliani G, Guerra F, Coletta D, Giuliani A, Salvischiani L, Tribuzi A (2022). Robotic versus conventional laparoscopic technique for the treatment of left-sided colonic diverticular disease: a systematic review with meta-analysis. Int J Colorectal Dis.

[49] Solaini L, Cavaliere D, Pecchini F, Perna F, Bazzocchi F, Avanzolini A (2019). Robotic versus laparoscopic right colectomy with intracorporeal anastomosis: a multicenter comparative analysis on short-term outcomes. Surg Endosc.

[50] Park JS, Kang H, Park SY, Kim HJ, Woo IT, Park IK, Choi GS (2019). Long-term oncologic after robotic versus laparoscopic right colectomy: a prospective randomized study. Surg Endosc.

[51] Li L, Zhang W, Guo Y, Wang X, Yu H, Du B, Yang X, Luo Y (2019). Robotic versus laparoscopic rectal surgery for rectal cancer: a meta-analysis of 7 randomized controlled trials. Surg Innov.

[52] Dalager T, Søgaard K, Bech KT, Mogensen O, Jensen PT (2017). Musculoskeletal pain among surgeons performing minimally invasive surgery: a systematic review. Surg Endosc.

[53] Dalager T, Jensen PT, Eriksen JR, Jakobsen HL, Mogensen O, Søgaard K (2020). Surgeons' posture and muscle strain during laparoscopic and robotic surgery. Br J Surg.

