# Supplementary material for: Robotic versus laparoscopic left colectomy: a systematic review and meta-analysis
Source: Int J Colorectal Dis. 2022 Jun 1;37(7):1497–507. doi: 10.1007/s00384-022-04194-8 (PMC9262793; doi:10.1007/s00384-022-04194-8)
Supplement: Supplementary file 1 — Supplementary file1 (DOCX 26 KB) [file 384_2022_4194_MOESM1_ESM.docx]

| Assessment of methodological quality | | | | | | | | | |
| --- | --- | --- | --- | --- | --- | --- | --- | --- | --- |
|  |  | **Selection** | | | | **Comparability** | **Outcome** | | |
| First author | **Total points** | **Representative cohort** | **Selection of controls** | **Ascertainment of exposure** | **Outcome not present at start of study** | **Comparable groups** | **Assessment of outcome** | **Follow-up duration** | **Follow-up complete** |
| Al Temimi | 7 | • | • | • | • | • | - | • | • |
| Kim | 8 | • | • | • | • | • | • | • | • |
| Gass | 8 | • | • | • | • | • | • | • | • |
| Bilgin | 8 | • | • | • | • | • | • | • | • |
| Xu | 8 | • | • | • | • | • | • | • | • |
| Casillas | 6 | • | - | • | • | • | - | • | • |
| Lim | 8 | • | • | • | • | • | • | • | • |
| Cassini | 8 | • | • | • | • | • | • | • | • |
| Beltzer | 8 | • | • | • | • | • | • | • | • |
| Maciel | 7 | • | - | • | • | • | • | • | • |
| Mlambo | 8 | • | • | • | • | • | • | • | • |
